# Familial Florid Cemento-Osseous Dysplasia: A Rare Manifestation in an Indian Family

**DOI:** 10.1155/2012/574125

**Published:** 2012-11-11

**Authors:** Adit Srivastava, Rahul Agarwal, Romesh Soni, Avesh Sachan, G. C. Shivakumar, T. P. Chaturvedi

**Affiliations:** ^1^Faculty of Dental Sciences, Institute of Medical Sciences, Banaras Hindu University, Uttar Pradesh, Varanasi 221005, India; ^2^Department of Oral Medicine and Radiology, UP Dental College, Uttar Pradesh, Lucknow 227105, India

## Abstract

Florid cemento-osseous dysplasia (FCOD) is one of the uncommon dysplasias affecting the maxillofacial region. The age group may vary from 19 to 76 years and typically presents in the 4th and 5th decades. In most cases patients do not have hereditary basis of disease, and only a few familial cases have been documented. As far as we know this is the 1st reported case of familial FCOD in an Indian family. The mother and son exhibited multiple sclerotic masses in both jaws. The mode of transmission appeared to be autosomal dominant with variable phenotypic expression.

## 1. Introduction

FCOD is a type of sclerosing lesion characterized by multiple exuberant lobulated densely opaque masses restricted to alveolar process in either or both jaws [1]. The term was first described by Melrose et al. in 1976. The word florid was introduced to describe the wide-spread, extensive manifestation of the disease [2]. FCOD is not associated with any other extragnathic abnormality, and there is no abnormality in blood chemistry of patient. The disease has a tendency for bilateral occurrence often symmetrically [3]. When the lesions are large, jaw expansion may be noted with dull pain or drainage in affected area. For asymptomatic patient, the best management consists of regular recall with prophylaxis and reinforcement of good home hygiene care. The management of symptomatic cases is more difficult. It should be differentiated from chronic sclerosing osteomyelitis [4].

FCOD is rare in Indian population, and less than 10 cases have been reported in literature, but no cases of familial FCOD in Indian family have been reported till now [4, 5].

## 2. Case Report

**Case 1**

**Figure 1:**
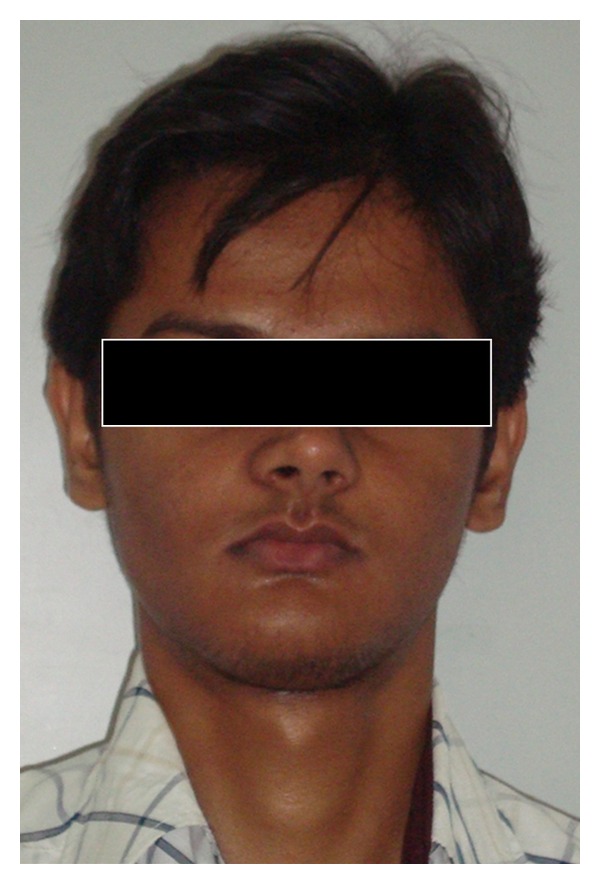
Extraoral photograph of patient (Case 1).

**Figure 2:**
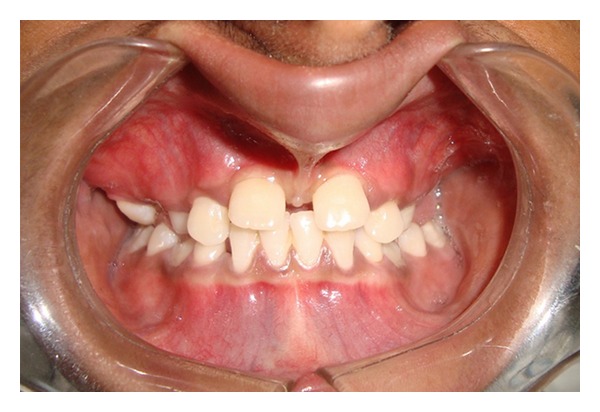
Intraoral photograph showing high frenal attachment (Case 1).

**Figure 3:**
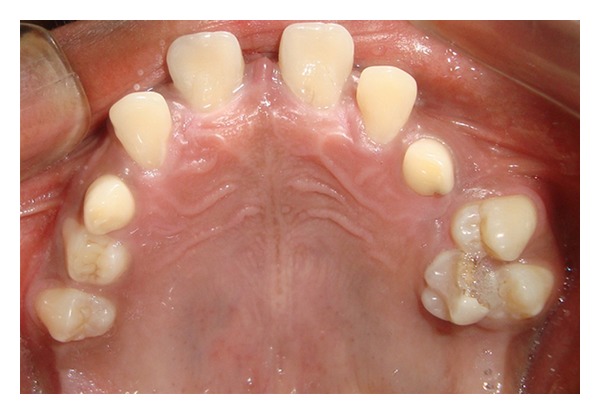
Intraoral photograph showing maxillary arch with retained deciduous teeth (Case 1).

**Figure 4:**
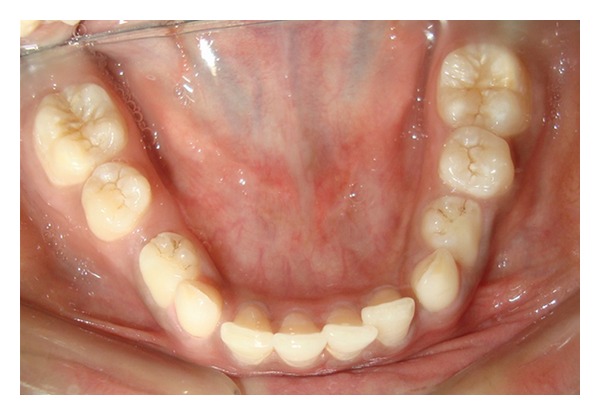
Intraoral photograph showing mandibular arch (Case 1).

**Figure 5:**
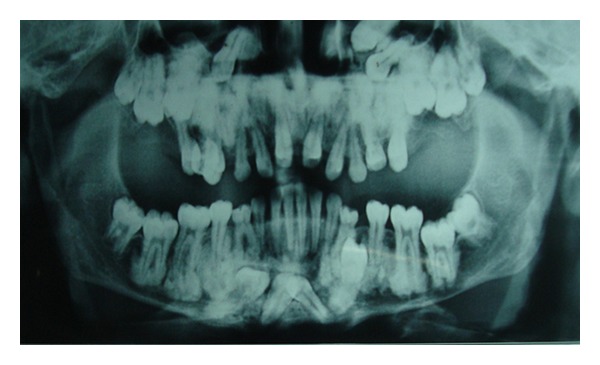
Panoramic radiograph (Case 1).

**Figure 6:**
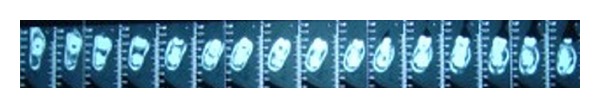
CT scan showing sections of lesion in the periapical region (Case 1).

**Figure 7:**
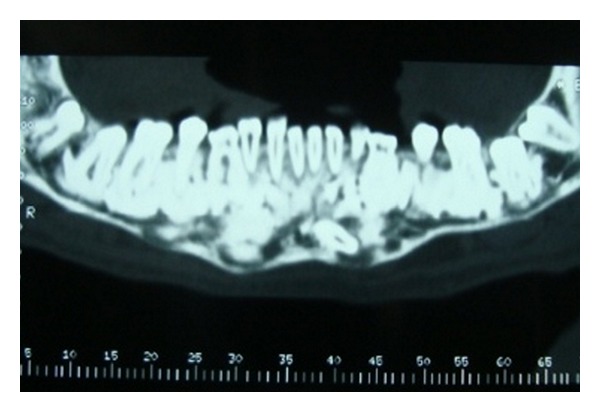
CT scan showing panoramic like reconstruction (Case 1).

**Figure 8:**
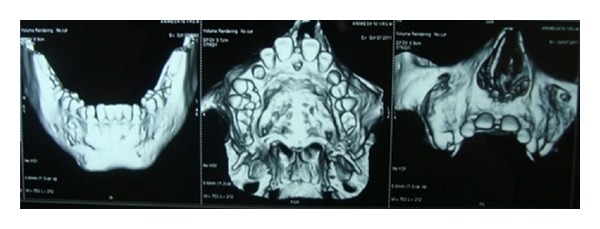
CT scan showing 3D reconstruction (Case 1).

A 18-year-old male patient (Figure 1) came to the Department of Oral Medicine, Faculty of Dental Sciences, IMS, BHU, with the chief complaint of spacing and malpositioned teeth and wanted to get it corrected. On extra-oral examination no abnormality was noticed except slight maxillary deficiency. Intraoral examination revealed high frenum attachment (Figure 2) between maxillary central incisors and missing 1st molar of both sides with retained maxillary deciduous second molar on the left side (Figure 3). All 4 canines were clinically missing. Slight buccal and lingual cortical plate expansion was apparent in mandibular molar region bilaterally (Figure 4). The patient was subjected to panoramic radiograph. OPG displayed a lobulated radio-opaque mass which was present in the apical region of almost all teeth in both maxilla and mandible in different stages of maturation. These masses were surrounded by ill-defined radiolucent lines (Figure 5). The patient was subjected to CT scan, and Denta-Scan programme was used. Axial sections of 1 mm thickness were obtained. The CT scan showed bone lesions adjacent to the root apices, and no cortical plate expansion was observed. Superior border of mandibular canal was intact. Panoramic like reconstruction demonstrated mesiodistal expansion of lesion and their relationship to the root apices. 3D reconstruction of mandible was done. Slight thinning of the cortical plate in the canine region was noticed (Figures 6, 7, and 8). Based on all these features a final diagnosis of FCOD was made, and under the differential diagnosis chronic sclerosing osteomyelitis and Paget's disease was considered.

Biochemical analysis of serum alkaline phosphatase, calcium and phosphorus was carried out to differentiate from Paget's disease and was found within normal limits.

**Case 2**

**Figure 9:**
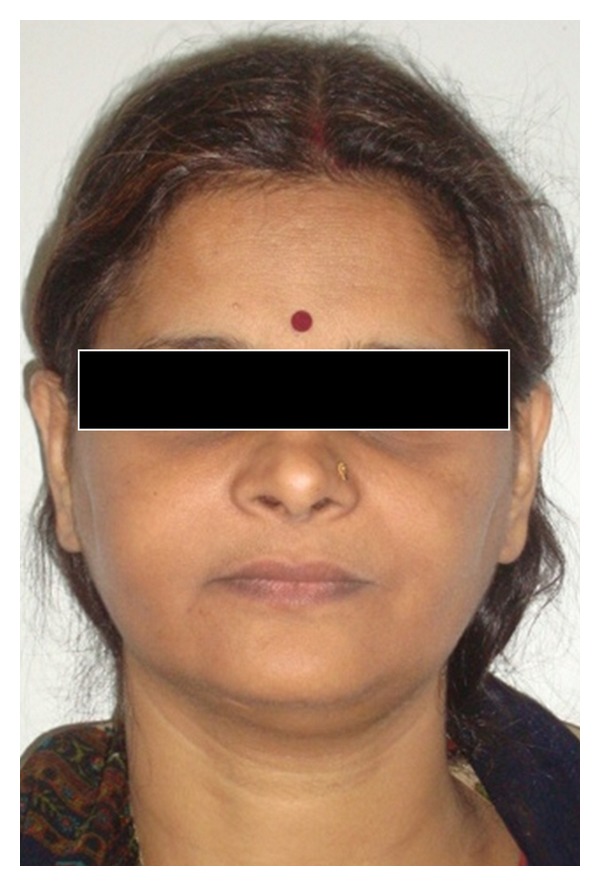
Extraoral photograph of patient's mother (Case 2).

**Figure 10:**
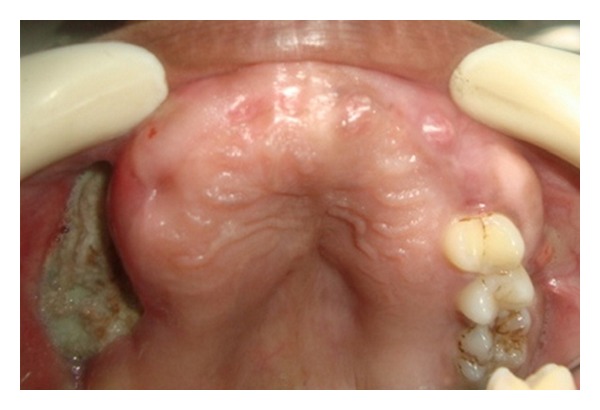
Intraoral photograph of patient's mother (Case 2).

**Figure 11:**
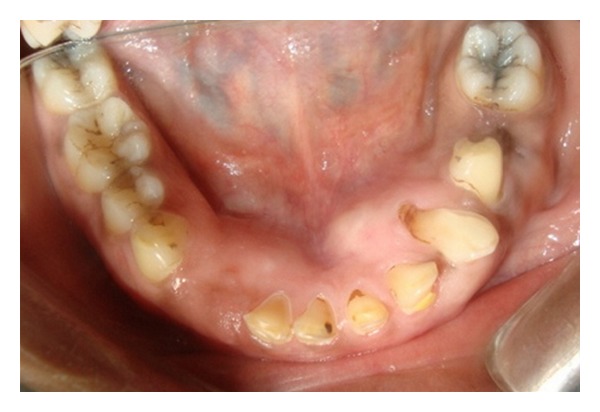
Intraoral photograph showing mandibular arch (Case 2).

**Figure 12:**
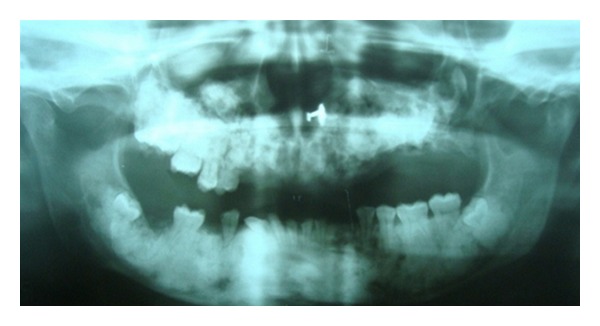
Panoramic radiograph (Case 2).

Patient reported that her mother also had similar problem. She had undergone a lot of treatment for the same problem (Figure 9). All the teeth of the maxilla were extracted except maxillary right premolar and molar region. In the region from maxillary left canine to 3rd molar region the bone was exposed in the oral cavity and was necrotic and covered by white slough (Figure 10). Mandibular left canine was missing, and slight buccolingual expansion was seen in incisor region (Figure 11). The patient was also subjected to OPG which has a similar appearance as her son (Figure 12).

**Figure 13:**
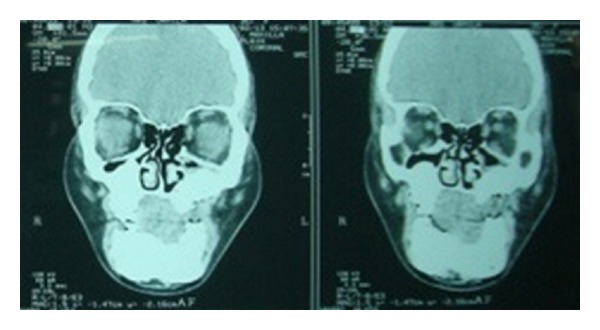
CT scan of patient's mother (Case 2).

**Figure 14:**
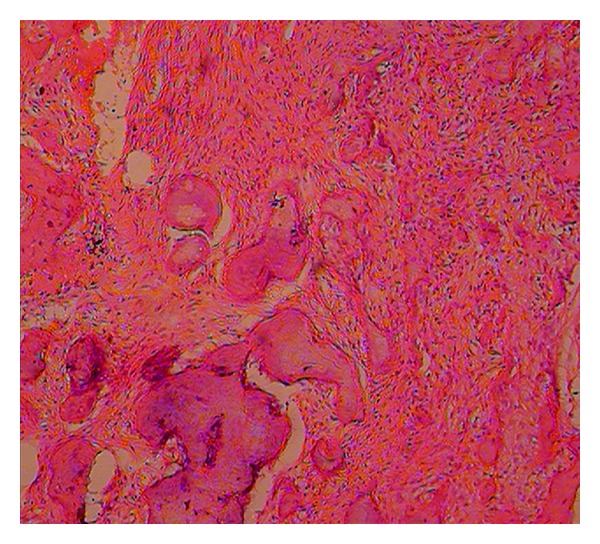
Photomicrograph (10x, H and E stained section) showing features of cemento-ossifying fibroma (Case 1).

She was also subjected to CT scan which showed radio-opaque masses scattered throughout mandible and maxilla (Figure 13). Radiographic features were similar to chronic sclerosing osteomyelitis. Patient also complained of pain in the exposed area. Surgical curettage was done, and surgical specimen was sent for histopathological confirmation (Figure 14). The biopsy report revealed it to be FCOD.

Based on the history, clinical feature, radiographic findings, and histological report a final diagnosis of familial FCOD was made. Both patients were educated about their disease and its course. As the son was asymptomatic, a periodic followup was advised. Both were advised to keep good oral hygiene. In mother's case surgical curettage was advised in the areas where she was experiencing pain as it would have been impractical to resect the lesion as it was occupying almost the whole of mandible and maxilla. Surgical intervention may lead to infection in the area which would have been very difficult to treat.

Surgical remodeling with curettage was done in mother's case. Antibiotics were also prescribed. Excised specimen was sent for histopathological confirmation, which confirmed our diagnosis. She became symptomless after one week. Both patients are under regular followup.

## 3. Discussion

The classification of cemento-osseous lesions of the jaws has long been matter of discussion for clinicians as well as pathologists. The current classification released by WHO in 1992 is based on age, sex, and histopathologic, radiographic, and clinical characteristics as well as location of lesion [6].

FCOD is the sclerotic lesion of the jaw characterized by diffused opaque masses limited to the alveolar process. In the past these calcifications have been interpreted as chronic diffused sclerosing osteomyelitis [7]. Chronic diffuse sclerosing osteomyelitis appears as a single, poorly delineated opaque segment of the mandible, whereas FCOD is seen as multiple round or lobulated opaque masses. Chronic diffused sclerosing osteomyelitis involves the body of the mandible from the alveolus to the inferior border and may extend into the ramus [8, 9].

Paget's disease of bone may also mimic this condition, only difference being that FCOD is centered above the inferior alveolar canal whereas Paget's involve the entire mandible [10].

FCOD is more commonly seen in middle-aged black women although it may also occur in Caucasians and Asians [1]. Though FCOD has the familiar autosomal dominant inheritance pattern, only a few cases have been reported. In our case a definite familial aspect of the disease could be established, and according to our knowledge it is the very first case of its kind to be reported in India [11–13]. The process may be totally asymptomatic, and in such cases the lesions are detected when radiographs are made for some other purpose [14]. Symptoms such as dull pain or drainage are almost always associated with exposure of sclerotic calcified masses in the oral cavity. This may occur as a result of progressive alveolar resorption under a denture or after extraction of teeth in the affected area [3, 13].

Radiographically, the lesion appears as multiple sclerotic masses located in two or more quadrants usually in the tooth-bearing area. They are often confined within the alveolar bone [15].

Histologically, these lesions are composed of anastomosing bone trabeculae and layers of cementum like calcifications embedded in a fibroblastic background [3, 5, 16].

Treatment of FCOD varies from case to case. Complete resection is generally impractical as lesion almost always occupied the entire mandible and maxilla. In asymptomatic patient it is wise to keep the patient under observation without surgical intervention. If required a remodeling resection is recommended for esthetic reasons [17].

The management of symptomatic patients is more difficult because chronic inflammation and infection develop within this densely mineralized tissues [18].

Antibiotics also have poor penetration in these areas and are not generally effective. In our case mother was having pain in the right upper back teeth region where the bone was exposed to the oral cavity [18].

## 4. Conclusion

Although clinical and radiographic findings were sufficient to establish the diagnosis in our case, histopathological examination was done in mother's case from the symptomatic area from where remodeling resection with curettage was done to relieve her pain. Familial cases are quite rare, and ours is the first familial FCOD to be reported in Indian literature.
